# Clinical Trial to Assess Physiology and Activity of Masticatory Muscles of Complete Denture Wearer Following Vitamin D Intervention

**DOI:** 10.3390/medicina59020410

**Published:** 2023-02-20

**Authors:** Shraddha Rathi, Saurabh Chaturvedi, Sabzar Abdullah, Geeta Rajput, Nasser M. Alqahtani, Mudita Chaturvedi, Vishwanath Gurumurthy, Ravinder Saini, Shashit Shetty Bavabeedu, Giuseppe Minervini

**Affiliations:** 1 Department of Prosthodontics, Crown & Bridge, Dr Z A Dental College, Aligarh Muslim University, Aligarh 202001, India; 2 Department of Prosthetic Dentistry, College of Dentistry, King Khalid University, Abha 62529, Saudi Arabia; 3 Assistant Professor and Clinical Director, Department of Oral and Maxillofacial Pathology, Private Practitioner, Bhopal 462001, India; 4 Department of Dental Technology, College of Applied Medical Sciences, King Khalid University, Abha 62529, Saudi Arabia; 5 Restorative Dental Sciences, College of Dentistry, King Khalid University, Abha 62529, Saudi Arabia; 6 Multidisciplinary Department of Medical-Surgical and Odontostomatological Specialties, University of Campania “Luigi Vanvitelli”, 80016 Naples, Italy

**Keywords:** masseter muscle, cholecalciferol, muscle performance, muscle strength, removable complete denture, ultrasonography, electromyography

## Abstract

*Background and Objectives*: Little information is available on the role of Vitamin D as a micro-nutrient deficiency with masticatory muscle efficiency and its effect on the function of removable prosthesis. The aim of this study was to evaluate the role of vitamin D on masticatory muscle activity among completely edentulous patients and its effect on the retention of removable complete dentures (RCDs). *Materials and Methods*: A non-randomized clinical control trial was conducted on completely edentulous patients (60.53 ± 7.01 years) in the Indian population between 2017 and 2019. Subjects were evaluated for temporomandibular disorders according to the Diagnostic Criteria for Temporomandibular Disorders (DC/TMD). Serum Vitamin D (S Vit D) levels, Ultrasonography (USG), and surface Electromyography (sEMG) readings of the masseter muscle were recorded at enrolment (Level 0), after 3 months of Vitamin D therapy (Level 3), and after consecutive 3 months of maintenance therapy, i.e., after 6 months from baseline (Level 6). The fabrication of new RCDs was done for all after the enrolment, and the retention of RCDs was assessed by asking a question regarding denture retention and asking respondents to mark their satisfaction on a 5-point Likert scale. Data were analysed using ANOVA, Paired’-test and Pearson correlation coefficients. A *p*-value less than 0.05 indicated a statistically significant association. *Results*: Between enrolment and a six-month follow-up, S Vit D levels showed an increase from 16.03 ± 5.68 ng/mL to 31.35 ± 9.28 ng/mL, showing an increase of 15.32 ± 9.38 ng/mL (95.57% rise). Statistically significant values were observed for USG and sEMG. *Conclusions*: Results showed that S Vit D affects masticatory muscle activity by improving its thickness and boosting its tonicity. Healthy muscles assist in the retention of RCDs, consequently aiding in mastication, speech, and phonetics, hence improving patient satisfaction. Clinical implication: Acknowledging the fact that the prevalence of Vitamin D deficiency is worldwide. We suggest Vitamin D therapy as a nutritional intervention among the elderly completely edentulous population, following dietary counselling, and consider Vitamin D therapy to be an adjunct to nutritional counselling for improving masticatory muscle activity and efficiency, which aids in RCD retention and stability. Consequently, improving oral health-related quality of life for individuals.

## 1. Introduction

Vitamin D is the hormone that regulates calcium phosphate homeostasis and mineral bone metabolism. Different tissues have varied receptors to absorb the micronutrient; vitamin D receptors are important as they are responsible for the biological effects of vitamin D [1,2,3,4,5,6]. Vitamin D deficiency is indeed extremely common in many chronic diseases, such as chronic obstructive pulmonary disease, cardiac insufficiency, cancer, and chronic kidney disease (CKD) [6], and the elderly population is most often affected by these problems. Vitamin D has been known to be of importance to musculoskeletal health, which is essential in old age [7,8].

Vitamin D is either consumed in the form of food or synthesized in the body when exposed to sunlight (accounting for 80–90% of the vitamin D body stores). Cholecalciferol (vitamin D3) is produced in the skin and found in fatty fish and mammals, while ergocalciferol (vitamin D2) is obtained from yeasts and plants. Vitamin D supplements contain vitamins D2 or D3, and recent studies have proved the superiority of vitamin D3 in the treatment of vitamin D insufficiency [9]. Vitamins D2 and D3 are both hydroxylated primarily in the liver to 25-hydroxyvitamin D (25OHD) and subsequently in the kidneys (and different peripheral cells) to become the active vitamin D metabolite, 1,25-dihydroxyvitamin D (calcitriol), which acts on the vitamin D receptor [10].

Generally, patients with vitamin D deficiency present with muscle pain and weakness [5,7,9]. According to SCAN, 2016 (Scientific advisory committee on nutrition), Vitamin D has a profound effect on oral health by regulating the rate of progression of bone loss during periodontal disease, which may modulate more rapid tooth loss in people with low serum cholecalciferol [6,9,10,11,12,13]. Replacement of missing teeth in completely edentulous patients is done most commonly by removable complete dentures (RCDs) [14,15]. Elderly denture wearers often find that their chewing ability is insufficient and that they are obliged to eat soft foods [1,16]. Edentulous patients find it fatiguing to disintegrate the food bolus into a digestible form [2,17,18], developing a dietary imbalance [3]. Reasons for eating soft food and the inability to break the food bolus into small pieces may be many, but retention of RCDs is one of the important factors. This results in the improper absorption of nutrients. The number of macronutrients (carbohydrates, proteins and fats) still can be absorbed from the larger bolus, but absorption of micronutrients becomes a challenge leading to nutritional deficiency. Consequently, several degenerative age changes occur in the body cells, making a person feeble.

Evidence shows that good muscle strength is a key to success in improving the retention of RCDs [4,19,20]. Patients with vitamin D (an essential micronutrient) deficiency present with muscle pain and weakness [5,21,22], which may affect the retention of the RCDs. Due to a deficiency in dietary intake of Vitamin D, supplements of it are advised orally or by injection [9]. Most experts consider Serum Vitamin D (S Vit D)- 25(OH)D of <20 ng/mL as deficient, and 21–30 ng/mL as insufficient [10,11,12,13]. Holick’s recommended [13] S Vit D levels > 30 ng/mL to take full advantage of all the health benefits.

Even in the modern era of dental implantology, a majority of edentulous patients still opt for RCD to improve oral health-related quality of life (OHRQoL) [23,24,25]. According to Jacobson and Krol, neuromuscular control is one of the key factors in providing retention as well as stability to a removable complete denture [4,26]. Craddock [5] described the gripping action of facial muscles on RCDs. The masseter influences the denture base during both the opening and closing movements of the mandible [23,27,28]. Consequently, it becomes important to evaluate the influence of facial muscle wasting on the retention and stability of RCDs [29]. Very little is known about the role of vitamin D on masticatory muscles and its impact on the denture-holding capacity of muscles, aiding in denture retention, thus making this study unique [30]. Therefore, the current research was conducted in the north Indian population of Aligarh Province as an interventional randomized control trial with the aim to evaluate the role of vitamin D on the masticatory muscle activity among completely edentulous patients and its effect on the retention of dentures as perceived by patients. The null hypothesis formulated in this research was that there would be no difference in the muscle activity of masticatory (masseter) muscles and patients would not perceive any change in retention of RCDs, after vitamin D therapy.

In the present study, the role of vitamin D on muscle activity was evaluated through surface electromyography (sEMG) [31,32,33,34,35,36] of the masseter muscle and its thickness, with the help of ultrasonography (USG) [36]. The values of sEMG and USG were compared before starting the vitamin D therapy and after the completion of the therapy. The retention of the RCDs as perceived by patients was assessed subjectively by asking related self-administered questions.

## 2. Materials and Methods

### 2.1. Study Design

A double-blinded, interventional, non-randomized control trial was conducted in the Aligarh Province (Uttar Pradesh, India) between 2017 and 2019. This study aimed to evaluate the role of vitamin D on masticatory muscle activity among completely edentulous patients and its effect on the retention of dentures as perceived by patients. The study was conducted in accordance with the Declaration of Helsinki, and the protocol was approved by the Ethics Committee of the Institute. JNMC, AMU, Aligarh, India [JNMC-AMU/ECL/22-2013-14]. The study protocol was developed, and all subjects gave their written informed consent for inclusion before they participated in the study.

### 2.2. Study Subjects and Sample Size

A total of 130 completely edentulous patients between the ages of 38 and 75 were recruited for the study. Subjects were evaluated for temporomandibular disorders according to the Diagnostic Criteria for Temporomandibular Disorders (DC/TMD) [37]. Inclusion criteria were asymptomatic subjects without any TMD, patients with no more than one year of edentulous state, and new denture wearer patients with high well-rounded residual alveolar ridges. Exclusion criteria considered were old denture wearer (more than 1 year), patients with any systemic disorders, drug allergies, on any immune drug therapy, weak thin, lean patients with possible muscle dystrophy, and patients in whom the loss of teeth occurred due to any trauma or bone fracture. All recruited patients underwent a S Vit D level assessment. Based on the S Vit D levels of the recruited subjects, these were categorized as subjects with adequate S Vit D (the control group) and subjects with deficient S Vit D amounts (the treatment group).

### 2.3. Methodology

In the present study, the assessments were done at three points in time: at 0 months (T0); 3 months (T3) and 6 months (T6).

Pre-intervention—At T0-S Vit D, USG and sEMG values were recorded for each enrolled subject. Based on S Vit D levels, only treatment group subjects were recommended the Vitamin D oral supplements.

Intervention: For treatment group subjects, the intervention of vitamin D was done orally following the recommendations of Holicks [33], Grant et al., and R. Vieth [38,39,40,41]. The Vitamin D was administered for 3 months till T3, and the maintenance dose was continued for the next 3 months till T6 in treatment group subjects.

At T3 and T6 the S Vit D levels, USG-measured muscle thickness and sEMG-measured muscle activity were recorded as compared with baseline (pre-intervention) T0. As far as treatment groups are concerned, they were formulated only to assess the need for vitamin D supplementation. The collected data comprised categorical variables as well as continuous variables. Therefore, the statistical tests were performed based on these two types of variables. The association of different clinico-demographic factors with S Vit D levels was analysed (Table 1). The collected variables were age, gender (male, female), occupation (farmer/labourer/field, skilled worker/priest, shopkeeper/service/business, professional, housewife/household), sun exposure (yes, no), eating habit (vegetarian, non-vegetarian), address (rural, urban), old denture (yes, no), S Vit D, USG measured muscle thickness, and sEMG measured muscle activity.

The data for S Vit D, USG-measured muscle thickness, and sEMG-measured muscle activity were collected at three different points in time: T 0 was the baseline after intervention, T3 was at 3 months, and lastly, the values of these three variables were collected at T6, which was at 6 months. The three values of S Vit D, USG measured muscle thickness, and sEMG measured muscle activity were paired values as they are the before and after values of an intervention.

At T0, even if the old dentures were present, for each subject they were removed for 1 week, and the recording of USG and EMG occlusal bite records were made (by the chief researcher and trained technician who was blinded from the study) for each patient, and the averaging of the EMG and USG values of masseter were completed in the resting position and during maximum bite clenching. After the recordings, the RCD fabrication and insertion were done for both groups of patients, following the normal conventional procedure for removable complete denture fabrication. In this procedure, initially, the primary impression was taken, and the primary cast was fabricated. Furthermore, after making a special tray, the secondary impression was made, and the master cast was poured with type IV die stone (GC Fujirock EP; GC Europe), to be used in further steps. Clinical steps involved were primary impressions, border molding with definitive impressions, jaw relations using a face bow [Hanau Spring-Bow (Whipmix, Louisville, KY, USA)] and inter-occlusal bite record with polyether bite registration material (RAMITEC, 3M ESPE, ST. PAUL, MN), teeth selection, wax trial placement, denture adjustment (expect occlusal surface adjustments), and insertion. Laboratory steps were master cast and special tray fabrication, mounting on a semi-adjustable articulator (Whipmix 2000 Serise-2240, Louisville, Ky, USA), teeth arrangement, and fabrication of CDs with conventional compression molding technique, lost wax technique, and a long polymerization cycle (9 h in a water bath at 73_C ± 1_C followed by 1/2 h in boiling water as recommended by the manufacturer) using heat-polymerizing acrylic resin (Dentsply Trubyte, INT. INC., York, PA, USA). The heat-polymerized CDs were finished and polished before the insertion visit [16,17,18,19,26]. Following the normal conventional protocol for complete denture insertion, patients were recalled after one week following their first recall visit. After one week, when patients reported, each patient was asked a question regarding denture retention (“What do you feel about the retention of your denture, during regular normal activities such as chewing, talking etc?”—Please mark your satisfaction on the Likert scale), as perceived by them and was asked to mark their satisfaction with denture retention based on a 5-point Likert scale as 5-highly satisfied; 4-satisfied; 3-cannot differentiate; 2-not satisfied, and 1-poorly retained [42].

At T3, after completing 3 months of drug regime and dentures use, all three readings (S Vit D, USG and sEMG) were recorded again for both group subjects. After assessing the values of S Vit D, the intervention of Vitamin D was continued as a maintenance dose for the next 3 months in treatment group subjects. All subjects were asked to continue denture use regularly. Furthermore, the same question regarding denture retention was asked, and the response was recorded on the same scale.

At T6, three months after T3, both the group control and treatment group subjects were recalled, and readings were taken for the same variables (S Vit D, USG, and sEMG) and the same question’ response was recorded.

#### Recording of S Vit D, USG, and sEMG

- Measurement of S Vit D

The institute’s medical pathology laboratory support was taken for the test. The trained technician blinded from the study collected the blood sample from each subject. The liquid chromatography-tandem mass spectroscopy (LC-MS) was applied for the direct measurement of 25(OH)D [25-hydroxyvitamin D] in the serum. The Vit-D levels were expressed in ng/mL [32,33].

- sEMG of masseter muscle

sEMG of the masseter muscle was done with surface electrodes, using surface electromyography apparatus (NeuroStim Ultra, NeuroStim Ultra series Machine, EMG-2000 series; Medicaid Systems, Mohali, Punjab; India) available in the institute pathology department. Surface electromyography (sEMG) is a form of measurement of the masticatory muscles’ functionality, able to identify variations of the electric potential of the muscles during each performed contraction, both in chewing and swallowing [34]. The technique used and the guidelines were previously explained to the patient. During recording, patients underwent mastication with their own cycles and spontaneous swallowing.

All sEMG tests were performed by the same technician as the chief researcher both of them had been calibrated for the same. Prior to each sampling, site friction with non-sterile gauze soaked in 70% alcohol was performed in order to minimize artifacts and improve signal capture. The reference electrode (earth) was placed in the front portion of the patient’s head. Data were obtained on the electrical activity of the masseter muscle group during the tasks of rest and maximum clenching (with maximum bite force, MBF). The records were collected by MBF, maintained for 5 s, and repeated three times with a 1 min interval addition to rest between each collection. The average was used for signal normalization, equivalent to 100% of the electrical activity. The signals collected during mastication were analysed by Root Mean Square (RMS) and expressed in microvolts (uV) [34,35,36].

- USG of Masseter muscle

All scans were carried out in the ultrasonography department. Each subject was examined by the same operator using the Thei Style Ultrasound system with a 7.5–9.0 MHz broadband transducer. A line was drawn joining the lateral commissure of the mouth to the intertragic notch (space that separates the tragus from the antitragus in the outer ear) of the ear, crossing the masseter muscle. A generous amount of water-soluble conductive gel was applied evenly on the muscle area on the cheeks using a gauze pad. The ultrasound probe was placed on the line with a feather-like pressure. The angle of the probe was adjusted to produce the strongest echo from the mandibular ramus, which was achieved when the scan plane was perpendicular to its surface. The imaging and measurements were performed bilaterally with the subjects in a supine position under two different conditions: when the teeth were gently occluding with the muscle in a relaxed position and during maximal clenching, with the masseter muscle contracted. The measurements were made directly from the image at the time of scanning [36].

Each measurement was recorded twice at an interval of 1 h and the average value was considered for analysis. The three levels of S Vit D, USG measured muscle thickness, and sEMG measured muscle activity was paired values as they are the before and after values of an intervention. A thorough analysis was conducted.

As far as the use of a repeated measures mixed model is concerned, we would like to admit that S Vit D levels over a period of 6 months are not dependent upon the vitamin D supplementation alone but are also dependent on a host of other factors such as season, dietary intake, level of sun exposure, etc., and hence it is not desirable to study this multifactorial model without considering all these factors.

### 2.4. Statistical Methods

Descriptive characteristics were determined for each variable. The mean ± standard deviation (sd) was reported for continuous variables, while the total frequency (percentage) was written for categorical variables. Pearson correlation and Spearman correlation coefficients were calculated for continuous and categorical variables. The correlation of S Vit D was estimated with each continuous and categorical variable. Apart from this, each level of S Vit D was tested for correlation with each level of USG-measured muscle thickness and sEMG-measured muscle activity. S Vit D was tested for significant differences with respect to the categorical variables gender, occupation, sun exposure, eating habit, address, old denture, and S Vit D status. Normality was tested for all continuous variables by using the Shapiro-Wilk test. If there were two categories, then independent samples *t*-test and Mann-Whitney tests were used depending on the normality assumption of the variables. For more than two categories, ANOVA with Scheffe post hoc and Kruskal-Wallis tests were applied to determine the statistical significance of the continuous variables between the groups of discrete variables. For pairwise comparisons after a significant Kruskal-Wallis test, the Mann-Whitney test was used with a Bonferroni correction. The three paired levels of serum D, USG-measured muscle thickness, and sEMG-measured muscle activity were analysed for significant differences using the Friedman test. The pairwise comparison after a significant Friedman test was made using a Wilcoxon signed rank test with a Bonferroni correction. The level of significance was fixed at 5%. The implemented statistical tests were said to be significant if the *p*-value was less than or equal to the level of significance. IBM-SPSS version 20 was used for conducting all the statistical analysis.

## 3. Results

A total sample of 130 patients was collected for the study, of whom 93 (71.5%) were males and 37 (28.5%) were females. The average age was 60.62 ± 6.94 years. The ratio of sun exposure was about the same, with the yes and no categories having 68 (52.3%) and 62 (47.7%) patients. Average S Vit D was 16.09 ± 5.62 (ng/mL) at level 0, 33.47 ± 10.83 (ng/mL) at level 3 and 31.31 ± 9.12 (ng/mL) at level 6. It is to be noted that the average values of S Vit D increased due to the intervention from level 0 to levels 3 and 6. Looking at the S Vit D status variable, it was found that there were no severely deficient patients at the 3-month and 6-month time points. The number of insufficient S Vit D individuals also reduced largely at 3 months and 6 months. From 3 months to 6 months, deficient S Vit D individuals increased. However, the number of patients slightly decreased in the sufficient S Vit D category. The Pearson correlation coefficient between level 0 S Vit D and level 0 USG measured muscle thickness was estimated at 0.611; between level 3 S Vit D and level 3 USG measured muscle thickness was 0.313; and between level 6 S Vit D and level 6 USG measured muscle thickness was 0.419. The highest correlation between S Vit D and sEMG-measured muscle activity was found between the two level 0 values (rp = 0.778). The descriptive characteristics in the form of mean ± sd, number (percentage), Pearson, and Spearman correlation are shown in Table 1.

Table 2 represents the Pearson correlation coefficients of S Vit D with USG-measured muscle thickness and S Vit D with sEMG-measured muscle activity. In Table 1, the correlations of these variables were only between level 0 with level 0, level 3 with level 3, and level 6 with level 6. However, Table 2 depicts all possible correlations between the three levels of vitamin D with three levels of USG-measured muscle thickness and three levels of S Vit D with three levels of sEMG-measured muscle activity. Starred values represent significance at a 5% level of significance.

The characteristics of S Vit D based on all categorical variables are represented in Table 3. Mean ± sd values are provided for each category of each variable. According to the Shapiro-Wilk test, level 0 and level 3 of vitamin D did not follow normal distribution (*p* value < 0.05) but level 6 values followed normal distribution (*p* value > 0.05). The average value of S Vit D at level 0 in males was 16.91 ± 5.58 (ng/mL) while in females it was 14.09 ± 5.23 (ng/mL). S Vit D at level 0 was statistically significant (*p* value < 0.05) between males and females. At level 6, the average value of S Vit D went up to 33.94 ± 10.09 (ng/mL) in males and 29.95 ± 9.74 (ng/mL) in females, and the results were statistically non-significant (*p* value > 0.05) between males and males. The occupation variable had 5 categories; therefore, Kruskal-Wallis and ANOVA tests were used. Only level 0 vitamin D values were significant (*p* value < 0.05). The significant difference was observed between farmer/labourer/field and housewife/household (*p* value < 0.01) and it was also observed between farmer/labourer/field and shopkeeper/Service/business (*p* value < 0.01). There was a significant difference (*p* value < 0.05) between the three levels of S Vit D continuous values and the three status variables of vitamin D. The remaining S Vit D characteristics based on categorical variables are presented in Table 3.

The two-sample analysis required to test the significance between the three levels of S Vit D continuous values and the three status variables of S Vit D is described in Table 4. The *p* values were obtained using a two-sample independent *t* test and the Mann Whitney test. These were followed by the application of the Bonferroni correction. At 3 months and 6 months’ time points, there were no severely deficient cases. Thus, no *p* values were estimated for such cases, and this is portrayed in Table 4.

Table 5 considers the comparison of vitamin D with all the continuous variables. The results obtained from applying the Shapiro-Wilk test to determine whether the variables follow a normal distribution or not are shown. Significant differences between the three levels of S Vit D and age, the three levels of USG-measured muscle thickness, and the three levels of sEMG-measured muscle activity were estimated by using two samples in an independent t test and the Mann Whitney test. The obtained results are shown in the table. The within-paired group analysis or three related samples analysis was tested by the application of Friedman test. The three levels of S Vit D were compared with each other. Similarly, the three levels of USG-measured muscle thickness were compared with each other, and lastly, a comparison was made among the three levels of sEMG-measured muscle activity. The achieved values for the Friedman test are depicted in the table. A comparison cannot be made with the same levels, and thus, the *p* values are not written. All three variables resulted in a significant *p* value (*p* value < 0.001) and it was concluded that there is a significant difference between the three levels of vitamin D. Similarly, it is concluded that a significant difference was determined between the three levels of USG-measured muscle thickness and between the three levels of sEMG-measured muscle activity.

**Figure 1:**
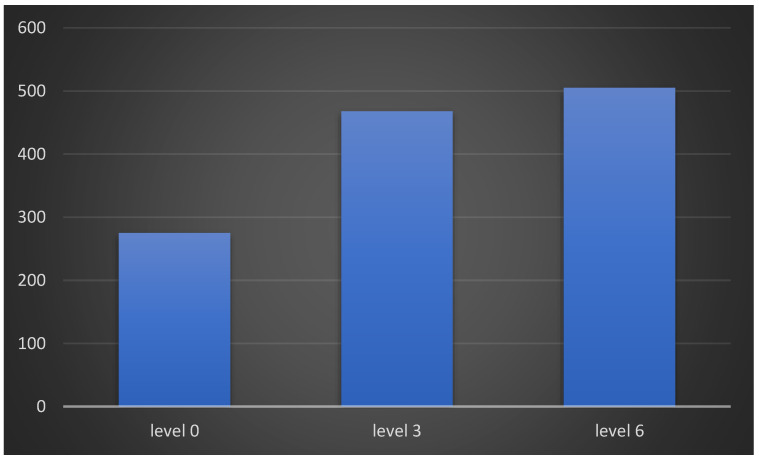
Maximum scores about responses for denture retention as perceived by patients at different levels.

To find out which paired levels had a significant difference, a pairwise comparison after the Friedman test was performed by using Wilcoxon signed rank test followed up with a Bonferroni correction. The resulting *p* values are presented in Table 6. It was concluded that there is a significant difference among all the levels of the three variables. In other words, it can be stated that there is a significant difference between the possible combinations of the three levels of S Vit D. Moreover, there is a significant difference between the possible combinations of the three levels of USG-measured muscle thickness and the possible combinations of the three levels of sEMG-measured muscle activity. Figure 1 shows the responses of patients to denture retention questions at different levels. The maximum score calculated for the Likert scale responses of 130 patients at level 0 was 275, at level 3 was 468, and at level 6 it was 505. As the S Vit D deficient patients received Vit D supplements there was an improvement in muscle activity and patients perceived related improvement in denture retention.

## 4. Discussion

In the present study, it was found that demographic and occupational variations have an impact on S Vit D levels [43]. Amongst the whole sample population, there were only 2.3% of S Vit D sufficient people; hence, it was proven that Vitamin D deficiency has become a nutritional pandemic. The present study also showed that rural-urban background variations affect vitamin D deficiency (Table 1). Modern-day studies have reported high rates of vitamin D deficiency even in groups of athletes from different parts of the world [36]. Factually, sun exposure is an untenable solution, for attaining cholecalciferol sufficiency. Low calcium intake in conjunction with Vitamin D deficiency makes things vulnerable. Consequently, the need for improvement in the vitamin D status of the Indian population is both important and urgent [44]. In accordance with the previous studies, the present study also did not find any significant variation in plasma Vit D levels based on their dietary intake. According to López-A D et al. [45], ethnic and social variation can be related to the lower intake of some food groups, but that does not show considerable changes in vitamin D status [46,47].

At enrolment, S Vit D levels ranged from 5.5 to 41.5 ng/mL in the study population. After intervention at 3 months, S Vit D levels range improved from 12.5 to 75.80 ng/mL. Initially, only 3 (2.3%) had S Vit D sufficiency, but after administrating Vitamin D therapy, we found the majority (63.1%) were vitamin D sufficient. After the intervention of 3 months, none of the patients had very severe S Vit D deficiency (Table 1). After a consecutive 3 months of maintenance therapy of cholecalciferol, vitamin D levels ranged from 12.0 to 60.5 ng/mL. The change occurred to 60.3% in the S Vit D sufficient group, and none of the subjects were severely deficient. As demonstrated by Żebrowska A et al., three weeks of Vitamin D supplementation had a positive effect on serum 25(OH)D levels in athletes [46,48].

In the present study, improvement was observed in the values of S Vit D, USG, and sEMG measured masseter muscle thickness and activity after 3-month intervention among group B subjects, which was in line with the study conducted by CegliaL [48] where Vitamin D supplementation was shown to improve tests of muscle performance and impact on muscle fibre composition in elderly patients. Geoffrey D. Abrams et al. [49] explained that vitamin D has a major role in muscle strength and performance.

In vitamin D insufficiency, the effect on muscle function and physical function mostly appeared before the clinical signs of bone disease were evident [50]; it had been reported in previous studies, meta-analyses, and even a clinical trial that there is an increased risk of falls in elderly subjects with low vitamin D levels, and if treated with vitamin D supplements, the risk of falls in the elderly is reduced [51,52]. Most likely, this is due to improved neuromuscular function in response to vitamin D supplementation. Similarly, the relationship between vitamin D status and muscle strength and physical performance has been studied in various cohort and cross-sectional studies [53,54]. Similar to these previous studies, in the present study, comparable observations were obtained after 3 months of the maintenance phase. There was a mild positive correlation between S Vit D levels and sEMG and USG values. Studies conducted by Bischoff et al. appear to show a relationship between 25(OH)D levels and various measures of changes in muscle strength and function with ageing, which was in association with the present study [55,56,57].

Regarding the association between S Vit D status and masticatory musculature efficiency at all three levels, after the intervention, both masseter muscle thickness and sEMG values showed a significant increase during the period (Table 1), thus indicating the sustainability of the masticatory efficiency achieved by the flowing Vitamin D intervention. Ceglia L et al. concluded that vitamin D supplementation improved muscle performance in vitamin D-deficient elderly patients. Therefore, the null hypothesis formulated was rejected as there was an improvement in muscle activity of the masseter muscles (confirmed by the results of USG and sEMG) and patients perceived and reported improved retention of RCDs, as affirmed by the high scores of responses of patients after vitamin D therapy. Henceforth, in the cohort of the completely edentulous population using RCDs, the role of Vitamin D therapy can play a key role in improving retention and stability.

The present study shows vitamin D as one of the most important factors affecting masticatory muscle activity, thus showing the need to include vitamin D assessment and supplementation as an important measure while planning the prosthodontic rehabilitation of the edentulous patient. However, it is the need of the hour to systematically evaluate the role of various other micro-nutrients on the various oral health-related issue at a global platform.

The present study had certain limitations, which include the short duration of the study. Furthermore, S Vit D levels are not only dependent upon Vitamin D supplementation only but are also dependent on a host of other factors such as season, dietary intake, level of sun exposure, etc. Similarly, the retention of dentures may also be affected by other factors which were not considered in the study, such as muscle activity and the quality of old dentures. The time and cause of tooth loss were not recorded, which might have affected the bony contour of the edentulous ridges. Furthermore, due to some reasons, muscle insertion might vary in a few patients, so it can be considered a physiological limitation. The effect of old dentures on ridges, soft tissues, and muscle activity was not evaluated in the study so as to simplify the study and establish a relationship between S Vit D levels and muscle thickness and activity, which play an important role in prosthodontics rehabilitation by RCDs. Thus, further studies are recommended to assess the need for long-term requirements of vitamin D therapy during the maintenance phase of deficiency-related ailments. Furthermore, the effect of various factors affecting RCDs including parameters such as edentulous ridge height, other nutritional deficiency, eating habits, education levels, etc., should be correlated in future studies for a better understanding of the role of vitamin D in denture retention. Along with all these, to avoid the Hawthorne effect in further studies, it is recommended to use either placebo tablets in the control group or administer vitamin D in the treatment group confined to dietary essentials.

## 5. Conclusions

Based on the findings of this non-randomized clinical trial, the following conclusions were drawn. The present study observed S Vit D deficiency irrespective of age, gender, or dietary habits. However, occupation, sun exposure, and a non-urbanization lifestyle had an impact on S Vit D levels. Only 2.3% of S Vit D-sufficient people were present in the study population, making it another nutritional pandemic. After administrating recommended Vitamin D therapy for three months, the majority (62.3%) became vitamin D sufficient. Vitamin D therapy has increased S Vit D levels resulting in improved USG and sEMG values of the masseter muscle, enhancing muscle thickness and boosting muscle activity, respectively. Acknowledging the fact that the prevalence of Vitamin D deficiency is worldwide. We strongly suggest vitamin D therapy as a nutritional intervention among the elderly completely edentulous population, following dietary counselling.

## Figures and Tables

**Table 1:** Descriptive Characteristics and Correlation with each time of recording of Vitamin D.

Variables	T0	T3	T6
Age	60.62 ± 6.94		
	r_p_ = 0.115	r_p_ = −0.052	r_p_ = −0.037
Gender Male Female	93 (71.5%) 37 (28.5%)		
	r_s_ = −0.263	r_s_ = −0.116	r_s_ = −0.126
Occupation Farmer/Labourer/field Skilled worker/priest Shopkeeper/Service/business Professional Housewife/household	47 (36.2%) 5 (3.8%) 49 (37.7%) 2 (1.5%) 27 (20.8%)		
	r_s_ = −0.388	r_s_ = −0.085	r_s_ = −0.096
Sun Exposure No Yes	62 (47.7%) 68 (52.3%)		
	r_s_ = 0.223	r_s_ = 0.037	r_s_ = 0.045
Eating Habit Vegetarian Non Vegetarian	90 (69.2%) 40 (30.8%)		
	r_s_ = −0.012	r_s_ = −0.053	r_s_ = −0.037
Address Rural Urban	71 (54.6%) 59 (45.4%)		
	r_s_ = −0.179	r_s_ = 0.013	r_s_ = 0.003
Old Denture No Yes	77 (59.2%) 53 (40.8%)		
	r_s_ = 0.244	r_s_ = 0.053	r_s_ = 0.105
Serum D (ng/mL)	16.09 ± 5.62	33.47 ± 10.83	31.31 ± 9.12
USG (mm)	6.53 ± 1.29	7.39 ± 1.21	7.59 ± 1.05
	r_p_ = 0.611	r_p_ = 0.313	r_p_ = 0.419
sEMG (μV)	151.21 ± 51.65	223.15 ± 52.81	248.91 ± 49.39
	r_p_ = 0.778	r_p_ = 0.424	r_p_ = 0.583
Vitamin D Status Severely Deficient Insufficient Deficient Sufficient	12 (9.2%) 94 (72.3%) 21 (16.2%) 3 (2.3%)	0 (0.0%) 11 (8.5%) 38 (29.2%) 81 (62.3%)	0 (0.0%) 11 (8.5%) 42 (32.3%) 77 (59.2%)
	r_s_ = 0.786	r_s_ = 0.856	r_s_ = 0.871

Mean ± standard deviation is represented for continuous variables and N (%) are depicted for categorical variables. ‘r_p_’ means Pearson correlation and ‘r_s_’ means spearman correlation.

**Table 2:** Correlation coefficients between Vitamin D and USG; Vitamin D and sEMG.

Vitamin D	USG			sEMG		
	Level 0	Level 3	Level 6	Level 0	Level 3	Level 6
Level 0	0.611 *	0.490 *	0.501 *	0.778 *	0.517 *	0.491 *
Level 3	0.160	0.313 *	0.355 *	0.155	0.424 *	0.558 *
Level 6	0.193 *	0.352 *	0.419 *	0.180 *	0.471 *	0.583 *

* Denotes that the correlation coefficients are significant at 5% level of significance.

**Table 3:** Testing Vitamin D with Categorical Variables.

	Vitamin D					
Variables	Level 0	Level 3	Level 6	*p* Value (Level 0)	*p* Value (Level 3)	*p* Value (Level 6)
Gender Male Female	16.91 ± 5.58 14.09 ± 5.23	33.94 ± 10.09 32.31 ± 12.58	31.87 ± 8.87 29.95 ± 9.74	0.003	0.187	0.281
Occupation Farmer/Labourer/field Skilled worker/priest Shopkeeper/Service/business Professional Housewife/household	18.89 ± 6.14 16.94 ± 3.65 14.59 ± 4.12 17.25 ± 2.05 13.75 ± 5.61	33.74 ± 7.87 32.20 ± 4.28 33.79 ± 11.63 44.35 ± 1.63 31.87 ± 14.41	31.81 ± 6.87 31.48 ± 3.41 31.54 ± 10.11 43.01 ± 0.42 29.19 ± 11.11	<0.001	0.219	0.301
				Farmer vs. Housewife (0.001) & Farmer vs. Shopkeeper (0.002)		
Sun Exposure No Yes	14.75 ± 4.63 17.34 ± 6.14	33.59 ± 12.51 33.37 ± 14.51	31.11 ± 12.01 31.51 ± 13.51	0.011	0.673	0.807
Eating Habit Vegetarian Non-Vegetarian	15.96 ± 4.86 16.43 ± 7.07	33.57 ± 9.62 33.26 ± 13.29	31.42 ± 8.43 31.12 ± 10.65	0.890	0.545	0.866
Address Rural Urban	17.07 ± 6.28 14.94 ± 4.45	33.62 ± 11.83 33.29 ± 9.59	31.43 ± 9.77 31.19 ± 8.38	0.042	0.879	0.888
Old Denture No Yes	14.95 ± 4.61 17.79 ± 6.49	33.25 ± 11.24 33.79 ± 10.31	30.81 ± 9.28 32.08 ± 8.94	0.005	0.547	0.435
Vitamin D Status (Level 0) Severely Deficient Insufficient Deficient Sufficient	8.11 ± 1.44 14.98 ± 2.81 22.72 ± 2.59 37.07 ± 4.67	20.38 ± 5.21 35.44 ± 9.81 31.51 ± 12.82 38.01 ± 3.77	20.51 ± 6.09 32.96 ± 8.41 29.33 ± 9.75 37.41 ± 3.35	<0.001	<0.001	<0.001
Vitamin D Status (Level 3) Severely Deficient Insufficient Deficient Sufficient	- 9.71 ± 2.71 16.17 ± 6.17 16.94 ± 5.08	- 16.06 ± 2.37 24.97 ± 3.04 39.82 ± 8.21	- 15.27 ± 2.82 24.47 ± 3.52 36.72 ± 6.33	<0.001	<0.001	<0.001
Vitamin D Status (Level 6) Severely Deficient Insufficient Deficient Sufficient	- 10.62 ± 3.25 16.42 ± 6.24 16.71 ± 5.13	- 16.55 ± 3.29 25.85 ± 4.24 40.05 ± 8.39	- 15.23 ± 2.72 24.74 ± 3.37 37.21 ± 6.14	0.001	<0.001	<0.001

**Table 4:** Summary of two samples analysis.

Vitamin D Status	Vitamin D	Severely Deficient vs. Insufficient	Severely Deficient vs. Deficient	Severely Deficient vs. Sufficient	Insufficient vs. Deficient	Insufficient vs. Sufficient	Deficient vs. Sufficient
Level 0 Status	Level 0	<0.001	<0.001	<0.001	<0.001	0.010	1.000
	Level 3	0.070	<0.001	0.019	0.087	0.654	1.000
	Level 6	<0.001	0.026	0.014	0.456	1.000	0.733
Level 3 Status	Level 0	-	-	-	<0.001	<0.001	1.000
	Level 3	-	-	-	0.172	<0.001	<0.001
	Level 6	-	-	-	<0.001	<0.001	<0.001
Level 6 Status	Level 0	-	-	-	0.002	<0.001	1.000
	Level 3	-	-	-	0.120	<0.001	<0.001
	Level 6	-	-	-	<0.001	<0.001	<0.001

**Table 5:** Testing Vitamin D with Continuous Variables and Three Related Sample Testing.

		Vitamin D			Friedman Test
Variables	Shapiro Wilk (*p* Value)	*p* Value (Level 0)	*p* Value (Level 3)	*p* Value (Level 6)	
Age	0.008	<0.001	<0.001	<0.001	
Serum D (Level 0)	0.000	-	<0.001	<0.001	<0.001
Serum D (Level 3)	0.001	<0.001	-	0.115	
Serum D (Level 6)	0.112 *	<0.001	0.115	-	
USG (Level 0)	0.001	<0.001	<0.001	<0.001	<0.001
USG (Level 3)	0.010	<0.001	<0.001	<0.001	
USG(Level 6)	0.155 *	<0.001	<0.001	<0.001	
sEMG (Level 0)	0.000	<0.001	<0.001	<0.001	<0.001
sEMG (Level 3)	0.335 *	<0.001	<0.001	<0.001	
sEMG (Level 6)	0.007	<0.001	<0.001	<0.001	

* Denotes that the variables follow normal distribution.

**Table 6:** Pairwise comparison after Friedman test and pairwise correlation.

	*p* Values and Correlation Coefficients		
Variables	Level 0 vs. Level 3	Level 0 vs. Level 6	Level 3 vs. Level 6
Vitamin D	<0.001	<0.001	<0.001
	r_p_ = 0.269 *	r_p_ = 0.292 *	r_p_ = 0.950 *
USG	<0.001	<0.001	0.001
	r_p_ = 0.926 *	r_p_ = 0.860 *	r_p_ = 0.945 *
sEMG	<0.001	<0.001	<0.001
	r_p_ = 0.645 *	r_p_ = 0.544 *	r_p_ = 0.889 *

* Denotes that the correlation coefficients are significant at 5% level of significance.

## Data Availability

Data can be made available on demand by the chief researcher for academic purposes by email.

## References

[1] Zarb G.A., Hobkirk J., Eckert S., Jacob R. (2012). Prosthodontics Treatment for Edentulous Patients.

[2] Ettinger R.L. (1973). Diet, nutrition, and masticatory ability in a group of elderly edentulous patients. Aust. Dent. J..

[3] Moynihan P., Petersen P.E. (2004). Diet, nutrition and the prevention of dental diseases. Public Health Nutr..

[4] Jacobson T., Krol A. (1983). A contemporary review of the factors involved in complete denture retention, stability, and support. Part I: Retention. J. Prosthet. Dent..

[5] Crescente G., Minervini G., Spagnuolo C., Moccia S. (2022). Cannabis Bioactive Compound-Based Formulations: New Perspectives for the Management of Orofacial Pain. Molecules..

[6] Guntona J.E., Girgisa C.M. (2018). Vitamin D and Muscle. Bone Rep..

[7] Atwood D.A. (1963). Postextraction changes in the adult mandible as illustrated by microradiographs of midsagittal sections and serial cephalometric roentgenograms. J. Prosthet. Dent..

[8] Chaturvedi S., Elmahdi A.E., Abdelmonem A.M., Haralur S.B., Alqahtani N.M., Suleman G., Sharif R.A., Gurumurthy V., A Alfarsi M. (2020). Predoctoral dental implant education techniques—students’ perception and attitude. J. Dent. Educ..

[9] Romagnoli E., Mascia M.L., Cipriani C., Fassino V., Mazzei F., D’Erasmo E., Carnevale V., Scillitani A., Minisola S. (2008). Short and Long-Term Variations in Serum Calciotropic Hormones after a Single Very Large Dose of Ergocalciferol (Vitamin D2) or Cholecalciferol (Vitamin D3) in the Elderly. J. Clin. Endocrinol. Metab..

[10] Hewison M., Zehnder D., Bland R., Stewart P. (2000). 1alpha-Hydroxylase and the action of vitamin D. J. Mol. Endocrinol..

[11] Scientific Advisory Committee on Nutrition (SACN) (2016). Vitamin D and Health. https://www.gov.uk/government/groups/scientific-advisory-committee-on-nutrition.

[12] Nair U.P., Shivamurthy R., Nagate R.R., Chaturvedi S., Al-Qahtani S.M., Al Magbol M., Gokhale S.T., Tikare S., Chaturvedi M. (2022). Effect of Injectable Platelet-Rich Fibrin with a Nano-Hydroxyapatite Bone Graft on the Treatment of a Grade II Furcation Defect. Bioengineering.

[13] Butera A., Pascadopoli M., Gallo S., Alovisi M., Lovati E., Mutti E., Scribante A. (2022). Domiciliary Management of Periodontal Indexes and Glycosylated Hemoglobin (HbA1c) in Type 1 Diabetic Patients with Paraprobiotic-Based Toothpaste and Mousse: Randomized Clinical Trial. Appl. Sci..

[14] Rajput G., Ahmed S., Chaturvedi S., Addas M.K., Bhagat T.V., Gurumurthy V., Alqahtani S.M., Alobaid M.A., Alsubaiy E.F., Gupta K. (2022). Comparison of Microleakage in Nanocomposite and Amalgam as a Crown Foundation Material Luted with Different Luting Cements under CAD-CAM Milled Metal Crowns: An In Vitro Microscopic Study. Polymers.

[15] Minervini G., Cervino G., Chaturvedi S., Franco R., di Francesco F., Fiorillo L., Cicciù M. (2023). Advanced method of rehabilitating edentulous Jaws: A review on telescopic denture. Technol. Health Care.

[16] Mittal P., Gokhale S.T., Manjunath S., Al-Qahtani S.M., Magbol M.A., Nagate R.R., Tikare S., Chaturvedi S., Agarwal A., Venkataram V. (2022). Comparative Evaluation of Locally Administered 2% Gel Fabricated from Lemongrass Polymer and 10% Doxycycline Hyclate Gel as an Adjunct to Scaling and Root Planing in the Treatment of Chronic Periodontitis—A Randomized Controlled Trial. Polymers.

[17] Cervino G., Montanari M., Santonocito D., Nicita F., Baldari R., De Angelis C., Storni G., Fiorillo L. (2019). Comparison of Two Low-Profile Prosthetic Retention System Interfaces: Preliminary Data of an In Vitro Study. Prosthesis.

[18] de Sire A., Ferrillo M., Gennari A., Cisari C., Pasqua S., Foglio Bonda P.L., Migliario M. (2021). Bone health, vitamin d status and oral hygiene screening in breast cancer women before starting osteoporosis treatment: A cross-sectional study. J. Biol. Regul. Homeost. Agents.

[19] Marenzi G., Spagnuolo G., Sammartino J.C., Gasparro R., Rebaudi A., Salerno M. (2019). Micro-Scale Surface Patterning of Titanium Dental Implants by Anodization in the Presence of Modifying Salts. Materials.

[20] Naddeo P., Laino L., la Noce M., Piattelli A., de Rosa A., Iezzi G., Tirino V. (2015). Surface biocompatibility of differently textured titanium implants with mesenchymal stem cells. Dent. Mater..

[21] Abouzeid H.L., Chaturvedi S., Abdelaziz K.M., Alzahrani F.A., AlQarni A.A.S., Alqahtani N.M. (2021). Role of Robotics and Artificial Intelligence in Oral Health and Preventive Dentistry—Knowledge, Perception and Attitude of Dentists. Oral Health Prev. Dent..

[22] Reddy L.K.V., Madithati P., Narapureddy B.R., Ravula S.R., Vaddamanu S.K., Alhamoudi F.H., Minervini G., Chaturvedi S. (2022). Perception about Health Applications (Apps) in Smartphones towards Telemedicine during COVID-19: A Cross-Sectional Study. J. Pers. Med..

[23] Shrestha B., Basnet B.B., Adhikari G. (2020). A questionnaire study on the impact on oral health-related quality of life by conventional rehabilitation of edentulous patient. BDJ Open.

[24] Chaturvedi S., Addas M.K., Alqahtani N.M., Al Ahmari N.M., Alfarsi M.A. (2021). Clinical analysis of CAD-CAM milled and printed complete dentures using computerized occlusal force analyser. Technol. Health Care.

[25] Cicciù M., Fiorillo L., D’Amico C., Gambino D., Amantia E.M., Laino L., Cervino G. (2020). 3D digital impression systems compared with traditional techniques in dentistry: A recent data systematic review. Materials.

[26] Chaturvedi S., Addas M.K., Alqahtani N.M., Al Ahmari N.M., Alfarsi M.A. (2021). Computerized occlusal forces analysis in complete dentures fabricated by additive and subtractive techniques. Technol. Health Care.

[27] Francesco D., di Francesco F., Lanza A., di Blasio M., Vaienti B., Cafferata E.A., Cervino G. (2022). Application of Botulinum Toxin in Temporomandibular Disorders: A Systematic Review of Randomized Controlled Trials (RCTs). Appl. Sci..

[28] Minervini G., del Mondo D., Russo D., Cervino G., D’Amico C., Fiorillo L. (2022). Stem Cells in Temporomandibular Joint Engineering: State of Art and Future Persectives. J. Craniofacial Surg..

[29] Iorio-Siciliano V., Blasi A., Stratul S.-I., Ramaglia L., Octavia V., Salvi G.E., Sculean A. (2021). Healing of periodontal suprabony defects following treatment with open flap debridement with or without an enamel matrix derivative: A randomized controlled clinical study. Clin. Oral Investig..

[30] Winkler S. (2015). Essentials of Complete Denture Prosthodontics.

[31] Minervini G., Mariani P., Fiorillo L., Cervino G., Cicciù M., Laino L. (2022). Prevalence of temporomandibular disorders in people with multiple sclerosis: A systematic review and meta-analysis. CRANIO^®^.

[32] Holick M.H. (2009). Vitamin D Status: Measurement, Interpretation and Clinical Application. Ann. Epidemiol..

[33] Byrd K. (1988). Loci and Characteristics of EMG Silent Periods During Masticatory Mandibular Movements in Rats. J. Dent. Res..

[34] Santos A.C., Silva C.A. (2016). Surface electromyography of masseter and temporal muscles with use percentage while chewing on candidates for gastroplasty. Arq. Bras. Cir. Dig..

[35] Ksiazek A., Zagrodna A., Słowińska-Lisowska M. (2019). Vitamin D, Skeletal Muscle Function and Athletic Performance in Athletes—A Narrative Review. Nutrients.

[36] Durão A.P.R., Morosolli A., Brown J., Jacobs R. (2017). Masseter muscle measurement performed by ultrasound: A systematic review. Dentomaxillofacial Radiol..

[37] Schiffman E., Ohrbach R., Truelove E., Truelove E., Look J., Anderson G., Ceusters W., Smith B. (2014). Diagnostic criteria for temporomandibular disorders (DC/TMD) for clinical and research applications. J. Oral Facial Pain Headache.

[38] Kimball M., Holick M.F. (2020). Official recommendations for vitamin D through the life stages in developed countries. Eur. J. Clin. Nutr..

[39] Grant W.B., Anouti F.A., Moukayed M. (2020). Targeted 25-hydroxyvitamin D concentration measurements and vitamin D3 supplementation can have important patient and public health benefits. Eur. J. Clin. Nutr..

[40] Vieth R. (2020). Vitamin D supplementation: Cholecalciferol, calcifediol, and calcitriol. Eur. J. Clin. Nutr..

[41] Limpuangthip N., Somkotra T., Arksornnukit M. (2019). Impacts of Denture Retention and Stability on Oral Health-Related Quality of Life, General Health, and Happiness in Elderly Thais. Curr. Gerontol. Geriatr. Res..

[42] Institute of Medicine, Food and Nutrition Board (2010). Dietary Reference Intakes for Calcium and Vitamin D.

[43] Gupta A. (2014). Vitamin D Deficiency in India: Prevalence, Causalities and Interventions. Nutrients.

[44] Díaz-López A., Jardí C., Villalobos M., Serrat N., Basora J., Arija V. (2020). Prevalence and risk factors of hypovitaminosis D in pregnant Spanish women. Sci. Rep..

[45] Żebrowska A., Sadowska-Krępa E., Stanula A., Waśkiewicz Z., Łakomy O., Bezuglov E., Nikolaidis P.T., Rosemann T., Knechtle B. (2020). The effect of vitamin D supplementation on serum total 25(OH) levels and biochemical markers of skeletal muscles in runners. J. Int. Soc. Sport. Nutr..

[46] Latham N.K., Anderson C.S., Reid I.R. (2003). Effects of vitamin D supplementation on strength, physical performance, and falls in older persons: A systematic review. J. Am. Geriatr. Soc..

[47] Ceglia L. (2009). Vitamin D and its role in skeletal muscle. Curr. Opin. Clin. Nutr. Metab. Care.

[48] Abrams G.D., Feldman D., Safran M.R. (2018). Effects of Vitamin D on Skeletal Muscle and Athletic Performance. J. Am. Acad. Orthop. Surg..

[49] Glerup H., Eriksen E.F. (1999). Acroparaesthesia—A typical finding in vitamin D deficiency. Rheumatology.

[50] Bischoff-Ferrari H.A., Borchers M., Gudat F., Dürmüller U., Stähelin H.B., Dick W. (2004). Vitamin D Receptor Expression in Human Muscle Tissue Decreases With Age. J. Bone Miner. Res..

[51] Jackson C., Gaugris S., Sen S.S., Hosking D. (2007). The effect of cholecalciferol (vitamin D3) on the risk of fall and fracture: A meta-analysis. QJM.

[52] Kuchuk N.O., Pluijm S.M.F., Van Schoor N.M., Looman C.W.N., Smit J.H., Lips P. (2009). Relationships of Serum 25-Hydroxyvitamin D to Bone Mineral Density and Serum Parathyroid Hormone and Markers of Bone Turnover in Older Persons. J. Clin. Endocrinol. Metab..

[53] Stewart J.W., Alekel D.L., Ritland L.M., Van Loan M., Gertz E., Genschel U. (2009). Serum 25-hydroxyvitamin D is related to indicators of overall physical fitness in healthy postmenopausal women. Menopause.

[54] Ward K.A., Das G., Berry J.L., Roberts S.A., Rawer R., Adams J.E., Mughal Z. (2009). Vitamin D Status and Muscle Function in Post-Menarchal Adolescent Girls. J. Clin. Endocrinol. Metab..

[55] Bischoff H.A., Stahelin H.B., Urscheler N., Ehrsam R., Vonthein R., Perrig-Chiello P., Tyndall A., Theiler R. (1999). Muscle strength in the elderly: Its relation to vitamin d metabolites. Arch. Phys. Med. Rehabil..

[56] Hamilton B. (2009). Vitamin D and Human Skeletal Muscle. Scand. J. Med. Sci. Sports.

[57] Ferrillo M., Nucci L., Giudice A., Calafiore D., Marotta N., Minervini G., de Sire A. (2022). Efficacy of conservative approaches on pain relief in patients with temporomandibular joint disorders: A systematic review with network meta-analysis. CRANIO^®^.

